# A Case of Vaginismus,—Treatment of, &c.

**Published:** 1867-05

**Authors:** 


*Great Northern Hospital.—A Case of Vaginismus; Treatment by Nitrate of Silver and Iodine; Recovery.* Under the care of Dr. G. C. P. Murray.

Some four years ago Dr. Marion Sims drew attention to an excessive super sensitiveness of the vagina in married women which he had observed in two or three instances. Accompanying this was an involuntary spasmodic closure of the mouth of the vagina, to which he proposed to apply the term "vaginismus."

Weh ave lately seen a case of this kind at the Great Northern hospital, in which Dr. Murray perfectly succeeded in removing the distressing condition. The patient was a woman of about thirty years of age, who had been married two years, but had never borne children. She described herself as suffering from excessive pain in and about the external genital organs. So sensitive were the parts that even the friction of her clothes was unbearable. The condition was constant, and had existed for several weeks before she applied for relief. Her appearance indicated anxiety and distress. Menstruation was regular, and she fancied that her symptoms were somewhat alleviated at the monthly periods. Since the commencement of her illness connexion had been absolutely impossible. Dr. Murray, on attempting to make a digital examination, was foiled owing to the excessive pain and spasm which were immediately produced. He could not succeed in introducing even the tip of his finger; the slightest pressure produced exquisite agony.

A tonic treatment with opiates was pursued, and a lead lotion ordered to be kept constantly applied to the vulva. No benefit resulting from these measures, in order to ascer tain accurately the condition of the parts, he placed her under the influence of chloroform, and had then no difficulty in introducing an ordinary-sized speculum. The mucous membrane of the vagina was much reddened, dry, and rough, with small papillæ prominent on its surface. The os uteri dresented on its posterior lip an ulcer, the size of a fourpenny-piece, and both lips were thickened as it by chronic inflammation. There was a thick phlegm-like discharge from the os, which was apparently retained in the highest part of he vagina by the spasmodic condition of the canal. So offensive had this discharge become by retention that it resembled that found in carcinomatous disease of the uterus. Dr. Murray, having thoroughly cleansed the parts, applied the solid nitrate of silver freely over the whole surface of the os and cervix uteri. He then introduced a fold of lint, saturated with a strong solution of nitrate of silver, into the speculum; and, when this was withdrawn, the lint was left in contact with the walls of the vagina. This was allowed to remain for about ten minutes before removal. This treatment was repeated on two subsequent occasions at intervals of a fortnight, when the patient was again under the influence of chloroform. After this, at her next visit, she was so much improved that no chloroform was required, and an application of diluted tincture of iodine was made to the os and cervix without occasioning her much distress. The discharge completely ceased, the ulcer healed, and the patient is now (about three months since the treatment was commenced) freed from the irritation which caused her so much misery.

In this case it is fair to connect the vaginismus with the diseased condition of the os uteri.—*Ib.*

